# Marine-Derived Polysaccharides and Their Potential Health Benefits in Nutraceutical Applications

**DOI:** 10.3390/md23020060

**Published:** 2025-01-28

**Authors:** Joana Carrasqueira, Susana Bernardino, Raul Bernardino, Clélia Afonso

**Affiliations:** 1 MARE—Marine and Environmental Sciences Centre/ARNET—Aquatic Research Network, School of Tourism and Maritime Technology, Polytechnic Institute of Leiria, 2520-614 Peniche, Portugal; joana.p.carrasqueira@ipleiria.pt (J.C.); susana.bernardino@ipleiria.pt (S.B.); raul.bernardino@ipleiria.pt (R.B.); 2 LSRE-LCM—Laboratory of Separation and Reaction Engineering-Laboratory of Catalysis and Materials, School of Technology and Management (ESTG), Polytechnic Institute of Leiria, 2520-614 Peniche, Portugal; 3 ALiCE—Associate Laboratory in Chemical Engineering, Faculty of Engineering, University of Porto, Rua Dr. Roberto Frias, 4200-465 Porto, Portugal

**Keywords:** nutraceuticals, marine-derived polysaccharides, bioactive compounds, antioxidant, anti-diabetic, gut microbiota regulation

## Abstract

Marine-derived polysaccharides have sparked immense interest in the nutraceutical industry as they possess a wide range of bioactivities which are highlighted in this review. These include antioxidants, anti-inflammatory, anti-cancer, gut microbiota regulator, anti-diabetic, and anti-obesity. Algae, marine invertebrates, vertebrates, and microorganisms are the main sources of marine polysaccharides, such as alginate, fucoidan, laminarin, carrageenan, chitosan, glycosaminoglycans, and exopolysaccharides. The structure and functional groups of these compounds influence their bioactive properties. Moreover, the functional properties of polysaccharides, such as gelling, thickening, and stabilising capabilities, are also crucial in product development, where they can serve as gluten substitutes in bakery goods and stabilisers in icings, sauces, and yoghurts. The potential of commercial products under development, such as marine polysaccharide supplements, is discussed, along with already commercialised products in the nutraceutical market. This review emphasises the enormous potential of marine-derived polysaccharides as bioactive compounds with health benefits and commercial value.

## 1. Introduction

According to the Food and Agriculture Organization (FAO), a sustainable diet is one that has a low environmental impact; respects biodiversity and ecosystems; and is economical, nutritious, and safe [1,2]. However, a growing concern, particularly in Western societies, is the incidence of obesity, cardiovascular disease, diabetes mellitus, cancer, and other diet-related diseases [3,4,5]. This is the result of a work-focused lifestyle that leads to frequent reliance on fast-food meals and neglect of physical activity [6]. For this reason, some consumers have recently become more concerned about their health and diet. As a result, demand for food enriched with nutritious ingredients or dietary supplements has increased. These contain bioactive molecules from natural sources, such as polysaccharides, peptides, phytosterols, and vitamins, that provide health benefits, including the prevention and treatment of certain diseases. This concept is called nutraceuticals, combining the words “nutrient” and “pharmaceutical” [5,7]. They are therefore great alternatives to ingredients known to be harmful to human health, such as saturated fats, salt, and sugar [4]. As a result, the markets for functional foods and nutraceuticals are growing steadily. In particular, the nutraceuticals market was valued at USD 712.97 billion in 2023 and is projected to grow at a compound annual growth rate (CAGR) of 8.4% from 2024 to 2030 [4,5,8]. Hence, given the potential of the functional food and nutraceutical industries, there is a continuous demand for health-promoting ingredients to improve their products [4].

Research into marine-derived ingredients such as nutraceuticals and dietary supplements has progressed significantly over the past years [9]. Nonetheless, the vastness and biodiversity of the oceans provide an endless resource for the discovery and investigation of new bioactive compounds or even new compounds altogether [7,10]. This process has been made possible by the significant advances in technology that allow for the extraction of marine resources that can be commercially exploited in a variety of industries [11]. The most common sources of bioactive compounds of interest are seaweed, marine invertebrates, vertebrates, and microorganisms. As consumers, especially in Europe and America, are sceptical about these products, there is still a long way to go to develop cost-effective, sustainable, healthy, and tasty products that are well accepted [7,12]. Marine organisms are richly filled with bioactive compounds due to the hostile environment. Biotic and abiotic factors such as salinity, pH, temperature, pressure, pollutants, and light exposure can fluctuate rapidly, and marine organisms must adapt to these environmental changes to survive. This results in the production of numerous primary and secondary metabolites in response to the surrounding environment, some of which are unique to the species [7,9,10,13,14,15,16]. These metabolites include polysaccharides, polyunsaturated fatty acids (PUFAs), polyphenols, pigments, proteins, enzymes, and vitamins [5,9,11]. Recent studies have shown that these bioactive compounds display biological and physiological activities such as anti-tumour, anti-diabetic, anti-inflammatory, antimicrobial, wound healing, immunomodulatory, and regulation of gut microbiota [4,7,9,11,13]. As a result, these bioactive compounds are widely used in the nutraceutical, pharmaceutical, and cosmeceutical industries [11,17].

Marine-derived polysaccharides have attracted attention as they are ubiquitous, abundant, essential for various biochemical processes and structural roles in marine organisms, and inexpensive to extract. Moreover, waste and by-products from the seafood industry can also be used, for example, in the development of dietary supplements or functional foods [4]. This not only contributes to food sustainability by promoting a circular economy but also promotes economic development. Polysaccharides can play distinct roles in organisms, e.g., chitin, alginate, and carrageenan have a structural role, whilst fucoidan is involved in algae’s defence mechanism [16,18]. Additionally, they also have a wide range of specific biological (e.g., anti-tumour, anti-diabetic, anti-inflammatory) and physicochemical (e.g., gelling, thickening, and stabilising) properties [9,16,19,20]. In fact, some polysaccharides such as agar (E 406) [21], alginate (E 400) [22], and carrageenan (E 407) [23] are widely commercially available as food additives and are generally recognised as safe (GRAS) [20]. These polysaccharides are also broadly used in the pharmaceutical and cosmetic industries, for example, in anticoagulants and anti-ageing creams, respectively [16,17]. Nevertheless, there are still several barriers to the commercialisation of nutraceuticals and functional foods, such as ensuring that the products meet consumer preferences and that there is sustainable evidence of the health benefits resulting from the consumption of the product [9].

Thus, this review explores the enormous potential of marine-derived polysaccharides as bioactive compounds that can be incorporated into nutraceuticals or functional foods, providing numerous health benefits.

## 2. Marine-Derived Polysaccharides

**Figure 1:**
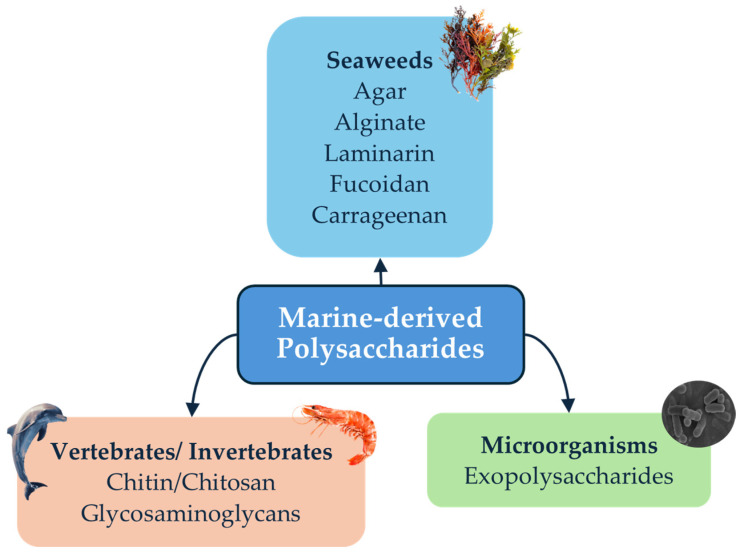
A few of the marine-derived polysaccharides from seaweeds, invertebrates, and microorganisms.

There are a wide variety of marine-derived polysaccharides, as described above, but in this review, we focus on those derived from macroalgae, marine invertebrates, vertebrates, and microorganisms, some of which are shown in Figure 1.

Polysaccharides derived from seaweeds account for 4 to 76% of their dry weight, depending on the species. These polysaccharides are located in the cell walls and intercellular space, allowing for great flexibility, which is pivotal for their adaptation to the aquatic environment [24]. These polysaccharides vary in their chemical and structural composition, as species, natural habitat, and even the seasons can influence their properties [25].

In marine invertebrates, polysaccharides are also present in substantial amounts and play a crucial role in protecting and supporting the organism, for example, through their presence in the exoskeleton [26,27]. In crustaceans, particularly, chitin and chitosan are the most abundant polysaccharides, namely in invertebrates’ shell walls [27]. Polysaccharides derived from these sources are usually used as drug delivery vehicles due to their physicochemical properties or otherwise in biotechnology and biomedicine as nanomaterials [28]. On the other hand, polysaccharides in marine vertebrates usually contribute mostly to the maintenance of the strength, elasticity, and hydration of various tissues. Glycosaminoglycans specifically are important in linking cells into tissues and then into organs, contributing to their overall structural integrity [29].

Polysaccharides derived from bacteria and fungi have become increasingly popular due to their ability to replicate quickly and to be easily manipulated [30,31]. These compounds display a wide variety of structures and therefore functions, as these are intrinsically linked, such as storage, structural, or protection from extrinsic factors [31]. These organisms can live in extremely hostile environments, and as a method of adaptation, they produce unique compounds, such as certain types of polysaccharides [31,32].

### 2.1. Brown Macroalgae Polysaccharides

The polysaccharides present in brown macroalgae have different properties from those of terrestrial plants, such as starch and cellulose [33]. The main naturally occurring polysaccharides in brown macroalgae are alginate, fucoidan (unique to this group of seaweeds), and laminarin [24,25].

#### 2.1.1. Alginate

**Figure 2:**
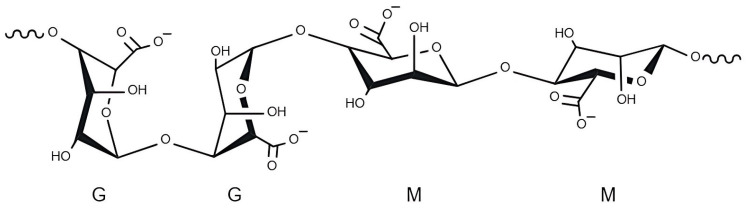
Chemical structure of alginate with G and M monomers.

Alginate was first identified by E.C.C. Stanford in 1881 [34]. This polysaccharide is characterised by the presence of β-D-mannuronic acid (M) and α-L-guluronic acid (G), which share a linkage in the β-(1,4) configuration. The positions of these monomers can change within the chain, resulting in the formation of different segments that are essentially either homogeneous (MM, GG) or heterogeneous (MG, GM) (Figure 2) [24,35,36]. The physical properties of alginate are determined by the presence of these three types of blocks in various proportions [24]. Alginates with a higher number of M-segments are more viscous, whereas alginates richer in G-blocks are more efficient gelling agents [36]. Alginate can be present in the form of alginic acid or as sodium and calcium salts and make up approximately 40% of the dry weight of the seaweed [24,36]. Alginate has a molecular weight (MW) of 500–1000 kDa [35]. The most common applications of this polysaccharide are in the cosmetic and food industries. In particular, it is widely used as a gelling agent, texturiser, and stabiliser in various types of products [24,36,37,38]. These properties can be attributed to alginate’s unique ion-binding characteristics, which enable it to selectively bind divalent metal ions, such as calcium [35,39].

#### 2.1.2. Fucoidan

**Figure 3:**
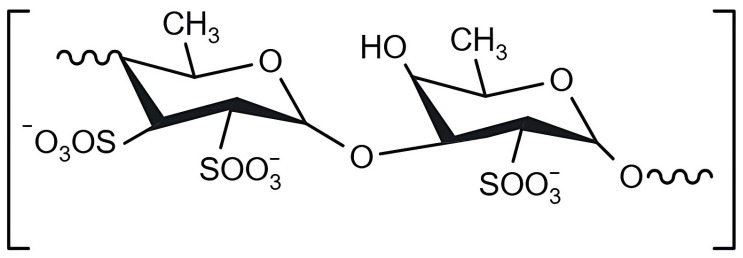
Chemical structure of fucoidan.

The polysaccharide fucoidan or fucose-containing sulphated polysaccharides (FCSPs) was originally named fucoidin by Professor Harald Kylin in 1913 [25]. As mentioned above, fucoidan can also be referred to as FCSP and is therefore characterised as a sulphated polysaccharide consisting of fucose, galactose, uronic acids, mannose, and xylose [25,35]. Fucoidan is composed of fucose linked predominantly by a backbone of α-(1,3) glycosidic linkages or repeating units of fucose residues linked by α-(1,3) and α-(1,4) bonds, with branching at the C2 position (Figure 3) [24,40]. These may also have sulphate esters at C4 or C2 of the main chain [40]. Nonetheless, the composition of these algal compounds varies from species to species and influences the overall structure of the polysaccharide [35].

Fucoidan represents 20 to 25% of the dry weight of macroalgae, depending on the seaweed species and environmental factors [41]. There is no consensus on the molecular weight of fucoidan, so it is believed that it can be established between 7 and 2300 kDa [24,35]. The role of fucoidan in seaweed is still not fully understood, but it has been reported that it may involve the prevention of dehydration and ensuring the structural stability of the macroalgae [40]. In terms of applications, fucoidan may be useful in the cosmeceutical and pharmaceutical fields, as it can act as a drug carrier or even improve the bioavailability of drug formulations, as is the case with nanoparticles [42].

#### 2.1.3. Laminarin

**Figure 4:**
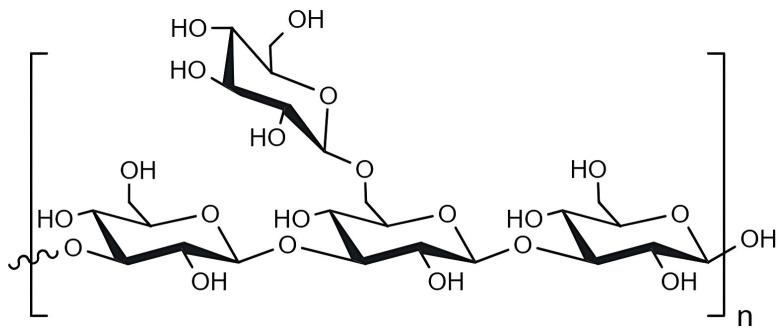
Chemical structure of laminarin.

Laminarin, also known as laminaran, was first identified in the brown macroalgae family Laminariaceae by Oswald Schmiedeberg in 1885 [25]. It has a molecular weight (MW) of approximately 5 kDa, depending on the degree of polymerisation, and is found in the vacuoles of seaweed [24,34,43]. Similarly, solubility can be influenced by the degree of branching. A laminarin with a high number of branches is soluble in cold water, whereas one with fewer branches is only soluble in warm water [35]. Laminarin consists of glucose units linked by β-(1,3) linkages with β-(1,6)-linked side chains (Figure 4) [24,25,35,43]. This polysaccharide may also contain a mannitol residue at the end of the chain [35,43]. The prevalence of this residue in the structure of laminarin depends on the species of seaweed and environmental factors, such as water temperature, salinity, sea current, and depth [25,43]. Laminarin is the main energy source in macroalgae, accounting for 22–49% of the dry matter in seaweed [25]. Seaweed uses it mainly during the winter after accumulating polysaccharides during the warmer seasons. One of the most relevant benefits of this compound is its ability to act as a dietary fibre, as the human organism is unable to digest it [38]. On the other hand, laminarin has been found to be a great asset in drug delivery and in the tissue engineering field as it can effectively support cell activities and promote tissue regeneration, which is particularly useful in the healing process [19].

### 2.2. Red Macroalgae Polysaccharides

Red macroalgae (Rhodophyceae) are exceedingly rich in polysaccharides, so their commercial value is mainly due to the use of these compounds in the food industry as emulsifiers and stabilisers as well as in the biomedical and pharmaceutical industries. Agar and carrageenan are undoubtedly the most abundant polysaccharides in this type of seaweed, accounting for 40–50% of the dry weight of the seaweed [44,45].

#### 2.2.1. Agar

**Figure 5:**
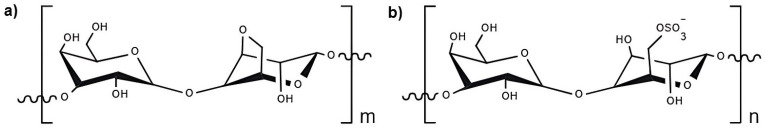
Chemical structure of (**a**) agarose and (**b**) agaropectin.

Agar was first discovered in Japan around the 17th century and is mainly found in the genera *Gelidium* and *Gracilaria*. Agar is responsible for algae’s structural elasticity, which allows it to withstand the force of the ocean currents [45,46]. This polysaccharide is a phycocolloid consisting of two different components: agarose and agaropectin, with agarose accounting for up to 70% of agar [45,47]. However, the proportion of agar, measured by weight, can vary between species, as can the agar content and the quality of the resulting gel, both of which are dependent on environmental factors [44,45,46,47]. Agarose is a neutral linear polysaccharide with an MW above 100 kDa and is composed of agarobiose units, i.e., β-D-galactose and 3,6-anhydro-L-galactose monosaccharides linked alternately by α-1,3 and β-1,4 glycosidic linkages (Figure 5). On the other hand, agaropectin is an acidic polysaccharide (MW of about 14 kDa), which, in addition to the agarobiose structure, can have ramifications such as sulphate groups in the C-6 position. Additionally, some β-D-galactose units may also contain pyruvic acid and D-glucuronic acid groups, thus preventing the polysaccharide from adopting a regular structure [21,44,48,49]. Hence, agarose is responsible for gelling properties due to the hydrogen bonds formed along its linear chain, whereas agaropectin has a thickening ability [46,47,48]. In terms of applications, agar is most used in the food industry as a gelling and stabilising agent, as it has already been granted GRAS status. Nevertheless, it is also widely used in the formulation of growth media in microbiology [44,49]. The human gastrointestinal tract is unable to digest this compound, and it has therefore been proposed for its use as a prebiotic [44,50].

#### 2.2.2. Carrageenan

**Figure 6:**
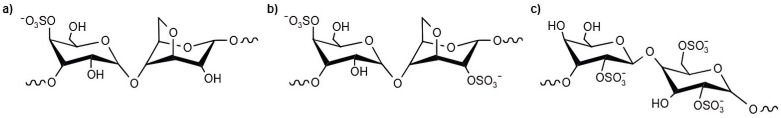
Chemical structure of (**a**) κ-carrageenan, (**b**) ι-carrageenan, and (**c**) ʎ-carrageenan.

Carrageenan is a polysaccharide extracted from red macroalgae commonly found along the coasts of Europe, North America, and Asia, such as in the Philippines [51,52]. This polysaccharide is a sulphated polygalactan with an ester-sulphate content of 15 to 40% and a high molecular weight of over 100 kDa. Carrageenan is composed of D-galactose and 3,6-anhydro-galactose units linked alternately by α-1,3 and β-1,4 glycosidic linkages. The most industrially interesting types of carrageenans are kappa (κ-) with one sulphate group, iota (ι-) with two sulphate groups, and lambda (λ-) with three sulphate groups per repeating disaccharide unit (Figure 6). This classification is therefore influenced by the number of ester sulphate groups and their position as well as the amount of 3,6 anhydro-D-galactose. These structural changes consequently lead to different physicochemical properties which determine the polysaccharide’s possible applicability [44,51,53,54].

Carrageenans are also considered to be GRAS, inexpensive, non-toxic, and biocompatible [44,53,54]. As such, they have a wide range of applications, including in the food, cosmetic, and pharmaceutical industries, such as gelling, thickeners, and stabilising agents [44,51,53,54]. Similar to agar and other polysaccharides, carrageenan is indigestible in the human digestive tract, and it can only be fermented by the colonic microbiota [44].

### 2.3. Marine Invertebrate and Vertebrate Polysaccharides

Marine invertebrates and vertebrates inhabit all marine ecosystems and are extremely diverse. Invertebrates make up most marine life and play a key role in their prosperity. They can be classified into the phyla Mollusca (oysters, clams, mussels, octopuses), Arthropoda (lobsters, crabs, shrimps), Echinodermata (sea stars and sea cucumbers), and Porifera (sponges) [13,27]. Vertebrates, such as marine mammals and reptiles, seabirds, fish, and jawless fish, are significantly less prominent but are still very diverse and remarkably complex organisms [55].

All these organisms serve as a rich source of countless important bioactive compounds, such as peptides, steroids, terpenoids, and polysaccharides [9,13]. These polysaccharides may have a structural purpose, as in the case of chitin and chitosan, which are prevalent in the exoskeletons of invertebrates [27]. On the other hand, they may also mediate cellular and physiological matters, contributing to cell growth, differentiation, and morphogenesis, as is the case with glycosaminoglycans [56,57].

#### 2.3.1. Chitin/Chitosan

**Figure 7:**
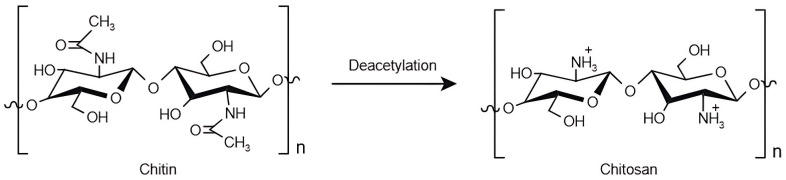
Chemical structure of chitin and chitosan after the deacetylation reaction.

Chitin is the second most abundant natural polymer in the world after cellulose and is synthesised by a large group of organisms to form their exoskeletons [9,58,59,60,61,62]. Chitin is composed of N-acetylated glucosamine units linked through a β-1,4 bond (Figure 7) [9,58,60,61,62,63]. It can be divided into three distinct groups: α-chitin typically extracted from the exoskeleton of crustaceans, β-chitin extracted from squid pens, and γ-chitin extracted from fungi and yeast [59]. Chitin is non-elastic and strong, forming an even stronger structure once it reacts to calcium carbonate (CaCO_3_), as in the exoskeleton of molluscs and crustaceans [63,64]. On the other hand, it is also hydrophobic and insoluble in most solvents due to its acetyl groups, so its applications are amplified when this compound is converted into chitosan [9,59,63]. Chitin can be converted to chitosan through a process of alkaline or enzymatic deacetylation in which the acetyl groups turn into hydroxyl (-OH) and amino (-NH_2_) groups [9,59,64]. As a result, chitosan consists of deacetylated and acetylated D-glucosamine units linked by β-1,4 bonds (Figure 7), with the degree of deacetylation usually determined by the ratio of glucosamine to N-acetyl glucosamine. Chitin is characterised as being richer in glucosamine than chitosan, which has a higher proportion of N-acetyl glucosamine [61,62,63]. The degree of acetylation can influence the chemical (e.g., tensile strength and solubility) and biological properties (e.g., bioavailability and biocompatibility) of these compounds [58,63]. Regarding the sources, chitin is mostly collected from crab and shrimp shells, which are often discarded and contribute to the waste of the marine industry. Extracting chitin and chitosan from these sources contributes to a circular economy, promotes sustainability, and reduces the environmental impact of marine waste [9,63]. Considering these polysaccharides’ properties, particularly their biodegradability, non-toxicity, and renewability, they can be used in a variety of pharmaceutical and biomedical applications. Other potential applications include wound dressings, food coatings, and tissue engineering [9,58,59]. Depending on the desired application, chitin and chitosan can take different forms, such as films, beads, membranes, gels, or sponges [61,63].

#### 2.3.2. Glycosaminoglycans

**Figure 8:**
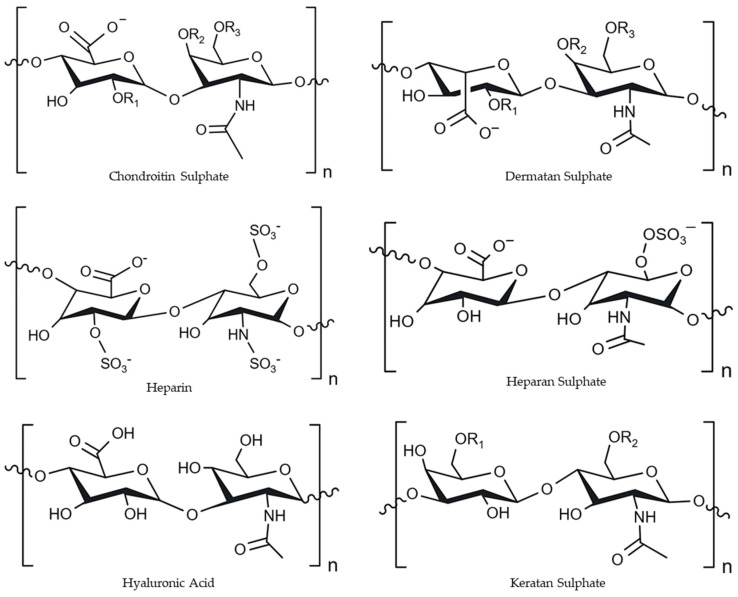
Chemical structure of the main GAGs in vertebrates (R_1_, R_2_, and R_3_ = H or SO_3_^−^).

Glycosaminoglycans (GAGs) are a complex family of polysaccharides that can be found in vertebrates, invertebrates, and even bacteria [57,65]. They are found in a wide variety of tissues, including blood vessel walls, lungs, liver, and skin tissues. They are essential for supporting cells, regulating osmotic pressure, and connecting cells and tissues [29]. GAGs from marine animals can differ significantly from those from terrestrial animals, with differences in the composition, degree of sulphation, and consequently biological properties [66]. GAGs can have an extended linear chain (MW 5–5000 kDa) negatively charged with repeating disaccharide units. The major GAGs in marine vertebrates include chondroitin sulphate (CS)/dermatan sulphate (DS), heparin (Hep)/heparan sulphate (HS), hyaluronic acid (HA), and keratan sulphate (KS) (Figure 8) [29,56,57,65,67,68,69]. These GAGs consist of modified units of amino sugars, such as N-acetyl-D-glucosamine (GlcNAc) or N-acetyl-D-galactosamine (GalNAc), and hexuronic acid (HexA), either iduronic acid (IdoA) or glucuronic acid (GlcA) [29,56,57]. These units are usually linked by 1,3-β or 1,4-β-glycosidic bonds [29]. Additionally, these compounds can undergo modifications by epimerisation and sulphation, which further contribute to their diversity and impact their biological activities [56,57]. GAGs can bind to proteins, forming proteoglycans, which can exert many beneficial biological effects, such as the regulation of cell proliferation and anticoagulant and antithrombin activities [56,65,67]. In fact, Hep has been used as an efficient anti-coagulant agent for more than 70 years, whilst HA, HS, and CS have been studied for their potential as dietary supplements in the treatment of osteoarthritis [56,67,70]. This shows that these polysaccharides have the potential to be used in the pharmaceutical and nutraceutical industries [65]. Furthermore, their properties may also be suitable for sectors such as tissue engineering, wound dressings, dermal fillers, and other biomedical applications [66].

### 2.4. Marine Microorganisms: Polysaccharides

The ocean is an exceedingly diverse and dynamic environment, and marine microorganisms are one of the main factors contributing to this richness. As in the case of algae, microorganisms can inhabit various environments, often with extreme conditions [71,72,73]. Consequently, they have developed special metabolic and physiological abilities to adapt to adverse conditions, such as the production of bioactive compounds, namely polysaccharides [72,73,74]. Microbial polysaccharides can be separated into three groups according to their location in the microbial cell: cell wall, intercellular, and exocellular polysaccharides (EPSs). EPSs are of particular interest due to their convenience of extraction in their pure form [73].

#### Exopolysaccharides (EPSs)

Marine microorganisms, particularly those living in extreme conditions, produce exopolysaccharides that are structurally and functionally diverse as a method of protection against environmental factors [9,74]. EPSs are polysaccharides with a wide range of molecular weights, which are secreted by several species of bacteria, fungi, archaea, and cyanobacteria. These polysaccharides can form a protective layer or otherwise enable the microorganism to adhere to certain surfaces, thus protecting it [9,73]. EPSs are structurally complex and typically consist of a combination of monosaccharides such as pentoses, hexoses, uronic acids, and amino sugars. If the backbone structure is more rigid, the monomers can be linked by 1,4-β- or 1,3-β-glycosidic bonds, and if it is more flexible, the monomers are typically linked by 1,2-α- or 1,6-α-glycosidic bonds [75]. Furthermore, these monosaccharides may also bind to proteins, lipids, or metabolites such as pyruvate, sulphate, acetate, and phosphate groups [76]. It has been reported that the monosaccharide composition, molecular weight, charge, and hydrophobicity of the EPS influence the type of protection required by different microorganisms, such as halophiles, psychrophiles, and thermophiles [74,77]. Several marine microbes that produce EPS have already been identified, including *Pseudoalteromonas*, *Bacillus*, *Alteromonas*, lactic acid bacteria (LAB) such as *Lactiplantibacillus plantarum* EI6, and Cyanobacteria [30,73,74]. Some examples of EPSs produced by bacteria are PEP, secreted by *Pseudoaltermonas* sp., which has anticancer properties [78], and EPS5SH, produced by *Bacillus* sp. H5, which has immunomodulatory activity [79]. Another bacterial EPS was first discovered in the species *Halolactibacillus miurensis*, called HMEPS, which has antioxidant activity that contributes to its survival in highly saline environments [80].

The marine fungi species *Keissleriella* sp., *Penicillium* sp., and *Aspergillus* sp. are also known to produce EPS with less diverse monosaccharides but with higher antioxidant activity when compared to bacteria [73,74]. The marine fungus *Penicillium solitum* is a producer of the EPS GW-12 [81], whilst *Aureobasidium melanogenum* SCAU-266 has been reported to produce the EPS AUM-1 [82].

In microalgae, there are several examples of species that produce EPS, for example, in the green microalgae *Tetraselmis suecica* [83] and *Chlamydonas reinhardtii* [84] and in the red microalgae *Porphyridium sordidum* [85].

The exopolysaccharides produced by microorganisms are particularly interesting because they have certain advantages over the polysaccharides synthesised by seaweed, plants, and animals. The main one is the fact that microorganisms can produce polysaccharides with high structural reproducibility, unlike most plants and seaweed, whose polysaccharide structures are dependent on environmental factors [73,86].

EPSs have gelling, thickening, adhesive, and stabilising properties that allow them to be used in a multitude of ways, particularly in the pharmaceutical, cosmetic, and food industries, and as a tool to combat environmental pollution (bioremediation and oil recovery) [9,72,73,74]. Regarding the application of EPSs in the food and nutraceutical industries, those derived from lactic acid bacteria (LAB) are already used in fermented food products as texturisers and present a longer effect in comparison to prebiotics already on the market. Moreover, *Lactobacillus* is considered GRAS, which facilitates the use of its products in the food industry. Due to the enormous diversity of marine microorganisms, it becomes a lengthy and complicated process to declare a new species as GRAS [73,87].

## 3. Biological Properties of Marine-Derived Polysaccharides

**Figure 9:**
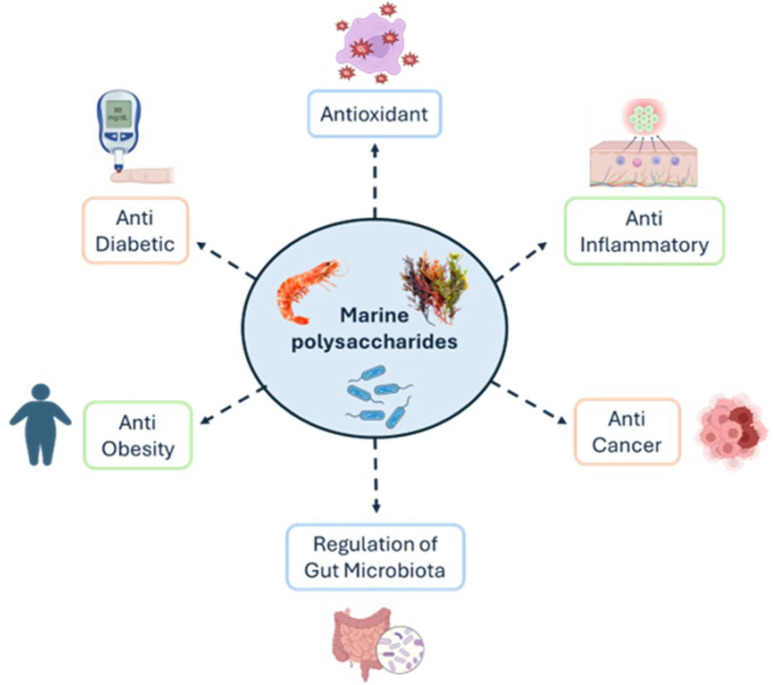
Overview of biological activities of marine-derived polysaccharides.

Marine-derived polysaccharides, such as the ones previously discussed, present unique structural features that contribute to their biological activities that include antioxidant, anti-inflammatory, anti-cancer, regulation of microbiota, anti-obesity and anti-diabetic (Figure 9). In this section, those key biological properties will be further explored.

### 3.1. Antioxidant

Every aerobic living organism has a metabolism that inevitably produces reactive oxygen species (ROS). Nevertheless, high levels of ROS might be harmful by destabilising the balance between oxidants and antioxidants, which can lead to oxidative stress [74]. Uncontrolled levels of ROS interfere with the normal activity of metabolites, such as lipids, proteins, and DNA, leading to the development of several diseases. These might include diabetes, cancer, and cardiovascular disease; however, it has been proven that antioxidants can have a preventative effect on these health problems [74,88]. Antioxidants are substances that inhibit oxidation by disrupting free radical chain reactions. They supply hydrogen molecules, stabilising free radicals so that they cannot initiate or promote further lipid oxidation or react with biomolecules [89].

It has been reported that marine organisms are rich in bioactive compounds that showcase antioxidant activity, namely polysaccharides [3,25]. Several research studies on the antioxidant activity of marine polysaccharides are listed in Table 1. There are several methods to assess biological activities, namely antioxidant activity, and they can take place in a living organism (in vivo) or in a cell, test tube, or Petri dish (in vitro). Animals such as mice are usually used in in vivo tests, where they are given a particular substance and later sacrificed and their blood and tissue samples analysed. On the other hand, several tests are commonly used for the in vitro method, such as DPPH, ABTS, ferric reducing-antioxidant power (FRAP), and superoxide radical scavenging activity (SOD) assays [90].

The effectiveness of polysaccharides as antioxidants depends on their structure, particularly their degree of sulphation, molecular weight, monosaccharide content, and the type of glycosidic bonds. These factors may influence the compound’s ability to donate hydrogen atoms to free radicals, for instance [9]. The antioxidant activity displayed by these compounds can generally be due to their ability to scavenge ROS, regulate the antioxidant system (i.e., regulation of SOD, catalase (CAT), and glutathione (GSH) levels), or even through oxidative stress-mediated induced pathways (e.g., SIRT1/AMPK/PGC1α and MAPK) [91].

When considering alginate, a study involving the development of low molecular weight alginates by thermal treatment showed a higher antioxidant activity when compared to an alginate polymer. This increase in activity was most likely due to the formation of additional functional groups. Therefore, antioxidants such as low molecular-weight alginates might be beneficial ingredients used in the food industry [92]. Fucoidan has been reported to prevent kidney damage due to oxidation by exerting antioxidant activity, which further suppresses the possible occurrence of kidney stones [93]. Jiang et al. showed that laminarin has antioxidant activity, as it was able to reduce the level of ROS formation in an in vitro culture of porcine embryos [94]. In another study, laminarin crude extract from the seaweed species *Laminaria hyperborea* had a higher antioxidant capacity than the purified version. Hence, it was concluded that even though laminarin is an antioxidant, its activity depends on the species of algae and the structure of the polysaccharide [95]. Agar was also found to have antioxidant properties in a study where food-grade quality agar from *Gracilaria tenuistipitata* was evaluated for its optimal extraction method and parameters. The alkali-extracted agar was found to have the best physiochemical and functional characteristics. Moreover, it is a low-cost extraction technology, making this agar a great option for incorporation into functional food products [96]. Carrageenan extracted from the red algae *Eucheuma gelatinae* had antioxidant activity and showed that it was directly proportional to the purity of the polysaccharide and inversely proportional to the increase in temperature [97]. Anraku et al. evaluated the effect of a high molecular chitosan supplement (HMCS), Chitosamin^®^, on the ROS levels in 10 individuals. The researchers noticed a decrease in the levels of lipid hydroperoxides and other uremic toxins and the suppression of further stimulation of oxidative stress in the systemic circulation. Thus, this supplement presents a particular antioxidant effect, which suggests that Chitosamin^®^ can aid in the treatment of more serious illnesses such as renal failure [98]. In one other study, chitin was extracted from the exoskeleton of a lobster (*Thenus unimaculatus*) shell and subsequently converted into chitosan, and its properties were evaluated. The results showed that chitosan has promising bioactivities, namely antioxidant activity, and can be used in the pharmaceutical industry as it is non-toxic [89]. GAGs have also been studied for their antioxidant activity, and one example of these studies is regarding GAGs extracted from the marine snail *Rapana venosa.* The authors concluded that this polysaccharide showed promising antioxidant activity, yet more detailed characterisation of the *R. venosa* GAG extract and in vivo testing is necessary to fully assess its potential as an antioxidant agent [67]. Finally, numerous studies have proven the antioxidant activity of exopolysaccharides. For example, Wu et al. showed that EPS273 produced by the marine bacterium *Pseudomonas stutzeri* 273 was effective against hydroxyl and superoxide anion radicals. In addition, it also showed anti-microbial abilities, making it a promising ingredient in food products [99]. Furthermore, Gongi et al. focused on the EPS produced by the filamentous cyanobacterium identified as *Leptolyngbya* sp. IkmLPT16. This exopolysaccharide displayed antioxidant activity through the capture of iron ions and scavenging of free radicals, hence it also has great potential for use in food applications [100].

**Table 1:** Summary of studies concerning the antioxidant activity of marine polysaccharides.

Bioactive Polysaccharides	Polysaccharide Source	MW	Models	Dose Period	Experimental Method	Results	Ref.
Alginate	_	50–250 kDa	In vitro	10^−4^–1 (*w*/*v*)	ABTS and SOD assays	Radical scavenging activity	[92]
Fucoidan	*Fucus* *vesiculosus*	_	Hyperoxaluric Wistar rats	5 mg/kg body weight, 28 days	Estimation of plasma malondialdehyde (MDA)	Decrease in MDA levels	[93]
Laminarin	_	_	Porcine early-stage embryo	20 μg/mL	Intracellular ROS levels and GSH assay	ROS scavenging activity, increase in GSH levels	[94]
Laminarin	*Laminaria* *hyperborea*	5.7–6.2 kDa	In vitro	1 mg/mL	DPPH and FRAP assays	Radical scavenging activity	[95]
Agar	*Gracilaria* *tenuistipitata*	_	In vitro	1–10 mg/mL	DPPH assay	Radical scavenging activity	[96]
Agaro- oligosaccharides	_	500–2500 Da	Human hepatocyte L-02	An array of concentrations (in vitro study)	Intracellular oxidant stress assay	Radical scavenging activity	[101]
			Mature Wistar rat	200–600 mg/kg, 10 days (in vivo study)	Biochemical assays	Inhibiting MDA, AST, and ALT	
Carrageenan	*Eucheuma* *gelatinae*	2.635 MDa and 2.58 MDa	In vitro	29.19 mg/mL	Total antioxidant and reducing power activity	Reducing power	[97]
Chitosan	_	100 kDa	Healthy individuals	540 mg/day, 2 months	Total plasma antioxidant capacity (TPAC), DPPH and ABTS assays	Increase in TPA; Radical scavenging activity	[98]
Chitosan	*Thenus* *unimaculatus*	_	In vitro	0.5–5 mg/mL	DPPH, ferrous ion chelating, and hydroxyl radical scavenging assay	Radical scavenging activity	[89]
GAG	*Rapana* *venosa*	_	In vitro	1–5 mg/mL	ABTS and FRAP assays	Radical scavenging activity	[67]
LSP and LMP	*Lapemis* *curtus*	84–89 kDa	In vitro	An array of concentrations	DPPH and ferrous ion chelating assays	Radical scavenging activity	[102]
EPS273	*Pseudomonas stutzeri* 273	190 kDa	In vitro	An array of concentrations	Hydroxyl radical and SOD	Radical scavenging activity	[99]
EPS	*Leptolyngbya* sp. IkmLPT16	_	In vitro	2–100 mg/mL	DPPH assay	Radical scavenging activity	[100]

### 3.2. Anti-Inflammatory

Inflammation is part of the biological response to noxious stimuli, such as infection, physical damage, or exposure to toxic substances. The inflammatory process is intricate and complex, involving several different pathways, enzymes, and transcription factors. While inflammation is part of the innate immune response and is therefore important, when it becomes excessive, it may cause extensive damage and even multiple diseases. The research for anti-inflammatory compounds and methods has, for this reason, gathered significant attention [18,103,104]. It has been assessed that the polysaccharide’s anti-inflammatory activity is heavily dependent on its MW, branching, backbone structure, and sulphation patterns [105]. Table 2 provides a summary of studies concerning the anti-inflammatory activity of marine polysaccharides.

Alginate is an example of a polysaccharide that showcases anti-inflammatory properties according to Jeong et al., who studied the effect of alginic acid on the mast cell-mediated anaphylactic and inflammatory response in in vivo and in vitro models. The results indicated that alginic acid successfully inhibited mast cell degranulator compound 48/80 (triggers systemic anaphylaxis shock) and significantly inhibited passive cutaneous anaphylaxis by 54.8%. Additionally, alginic acid suppressed histamine release from serum and peritoneal mast cells and the secretion and mRNA expression of pro-inflammatory cytokines such as IL-1 and TNF-α. Hence, the authors concluded that alginic acid can be used as an aid in the treatment of anaphylactic and inflammatory diseases through a variety of strategies [106]. Fucoidan extracted from *Saccharina japonica* contributed to the reduction in oxidative stress, inflammation, DNA damage, and inflammatory biomarkers (IL-1, IL-6, and TNF-α) in rats with diabetic mellitus [107]. Rattigan et al. studied the effects of laminarin on colonic health in pigs with dextran sodium sulphate (DSS) and found that it had anti-inflammatory potential and could be used as a dietary supplement in the treatment of inflammatory bowel diseases (IBDs) [108]. Nevertheless, not all polysaccharides derived from seaweed possess anti-inflammatory activity, as is the case with carrageenan, which is often used as a pro-inflammatory agent in the rat paw oedema model, a test that evaluates the efficacy of anti-inflammatory drugs [109].

Concerning polysaccharides derived from invertebrates, chitosan’s effects on ulcerative colitis (UC), a type of IBD, have been studied. The researchers tested different-sized chitosans in the LPS-induced Raw 264.7 cell model and the DSS-induced UC mice model. The results indicated that the size of the molecule was directly proportional to its effect on the disease. The therapeutic effects observed consisted of anti-inflammatory activity and regulation of the intestinal microbiota by inhibiting the release of pro-inflammatory cytokines, decreasing oxidative stress levels, and restricting the presence of certain microorganisms [110]. A sulphated glycosaminoglycan (PVP-2) extracted from the Asian green mussel *Perna viridis* has also been studied, both in vitro and in vivo, for its potential as an anti-inflammatory agent. This compound inhibited both cyclooxygenases (COX-1, 2) and 5-lipoxygenase (5-LOX) and reduced NO levels in LPS-activated Raw 264.7 cells. Moreover, in vivo experiments attested to the anti-inflammatory activity, as carrageenan-induced paw oedema in mice was significantly attenuated with the ingestion of PVP-2. These results clearly show the potential of PVP-2 extracted from *Perna viridis* to be used for the treatment of inflammation-related diseases [111]. In terms of the anti-inflammatory activity of exopolysaccharides, Hafez et al. conducted a study regarding the biological activities of marine *Pseudoalteromonas shioyasakiensis* SE. It was concluded that its EPS (EPSSE) could suppress COX-1 and COX-2, with greater efficacy against COX-1. These results suggest the potential use of this compound as an anti-inflammatory drug [112].

**Table 2:** Summary of studies concerning the anti-inflammatory activity of marine polysaccharides.

Bioactive Polysaccharides	Polysaccharide Source	MW	Models	Dose, Period	Experimental Method	Results	Ref.
Alginate	*Macrocystis* *pyrifera*	_	Rat peritoneal mast cells	0.01–1 mg/ mL (in vitro study)	Histidine decarboxylase and interleukins-1β, 6, 8, and tumour necrosis factor-α secretion assays	Inhibited histamine release, IL-1β, and TNF-α	[106]
			Male Wistar rats	0.25–1 g/kg (in vivo study)	Compound 48/80-induced systemic anaphylactic shock; Passive cutaneous anaphylaxis	Inhibited compound 48/80, inhibited passive cutaneous anaphylaxis	
Fucoidan	*Saccharina* *japonica*	_	Streptozotocin-induced diabetic rats	100 mg/kg/day, 2 months	Determination of inflammatory biomarkers	Reduced IL-1β, IL-6, and TNF-α levels	[107]
Laminarin	*Laminaria* spp.	_	Pigs challenged with dextran sodium sulphate (DSS)	200 ppm, 35 days	Colonic gene expression	Alterations in the pattern of co-expressed genes	[108]
Agaro- oligosaccharides	_	_	Colitis-induced C57BL/6 mice	4 g/kg/day, 6 days	Measurement of myeloperoxidase activity (MPO); Immunosorbent assay of tumour necrosis factor-α	Induced HO-1 expression; Suppressed TNF-α, IL-12, and IL-17A expressions	[113]
Chitosan	_	3–200 kDa	LPS-induced Raw 264.7 cells	0–1600 μg/mL (in vitro study)	Determination of NO, IL-6, and TNF-α in the supernatant of the cells	Decreased the levels of NO, IL-6, and TNF-α	[110]
			DSS-induced UC mice	150 mg/kg and 300 mg/kg, 10 days (in vivo study)	Determination of IL-6, TNF-α, IL-1β, IL-10, and IgG in serum of mice	Decreased inflammatory cell infiltration and IL-6, TNF-α, IL-1β, IL-10, and IgG levels	
PVP-2	*Perna viridis*	_	LPS-induced Raw 264.7 cells	1–10 μg/mL (in vitro study)	COX and 5-LOX inhibition assays	Decreased the levels of COX 1 and 2, 5-LOX, and NO in the cells	[111]
			Carrageenan-induced paw oedema mice	22–110 mg/kg body weight (BW), 10 days (in vivo study)	Determination of NO Effects of PVP-2 in carrageenan-induced paw oedema	Reduced paw oedema in mice	
D-SBSG	*Aristichthys* *nobilis*	4.96 kDa	Raw 264.7 cells	0.25–1 mg/mL	NO and cytokine secretion assays ROS generation assay	Decreased the levels of NO, IL-6, IL-1β, IL-10 and ROS	[114]
EPSSE	*Pseudoalteromonas shioyasakiensis* SE	8 kDa	In vitro	0.01–100 μg/mL	*N*, *N*, *N*′, *N*′-tetramethyl-p-phenylenediamine (TMPD) assay method	Inhibited COX-1 and COX-2	[112]

### 3.3. Anti-Cancer

Cancer consists of an array of diseases involving abnormal cell growth, promotion of angiogenesis, evasion of growth suppressors, resistance to cell death, and stimulation of invasion and metastasis. Cancer may be caused by DNA damage, oxidative stress, and inflammation and is currently a leading cause of death worldwide. Given its prevalence, the search for alternative treatment and prevention methods has significantly increased, specifically by resorting to marine-derived natural products. This is due to the acknowledgement that in East Asia, cancer is typically less prevalent than in Western societies, and its natives are known to have incorporated seaweeds and other marine organisms into their diets [115,116].

Several studies have highlighted the anti-cancer effects of marine-derived polysaccharides. These compounds may exhibit direct anticancer activity by inducing tumour cell apoptosis, inhibiting tumour angiogenesis, and having an immunoregulatory effect, or they may serve as drug delivery agents (Table 3) [117,118]. Among these polysaccharides, alginate has been explored for its potential in drug delivery applications. For instance, in one study, Ca-alginate beads were infused with apigenin (AGN), a natural flavonoid, and a significant reduction in the viability of MCF-7 breast cancer cells was observed. The apigenin-containing beads showed greater effectiveness against these cancer cells compared to apigenin alone. In addition, the incorporation of AGN into alginate beads increased its solubility and bioavailability, proving to be efficient microcarriers with anti-cancer activity [119]. Laminarin has also been studied as a drug-delivery agent by incorporating it into silver nanoparticles (AgNPs) to enhance its biomedical properties. These nanoparticles were shown to inhibit Y79 retinoblastoma cell lines by inducing apoptosis, as evidenced by DNA fragmentation, later detected by a DNA ladder assay [120]. Similarly, Cui et al. developed selenium nanoparticles containing laminarin, which also had anti-cancer effects in HepG2 liver cancer cell lines [121].

Beyond alginate and laminarin, other marine-derived polysaccharides have also shown promising anti-cancer properties. As is the case of fucoidan extracted from the seaweed *Sargassum cinereum*, which inhibited the growth of Caco-2 cells causing apoptosis through the release of ROS and an increase in mitochondrial membrane permeability, proving to be a promising anti-carcinogenic agent [122]. George et al. proved the anti-cancer effect of agar extracted from *Laminaria digitata* on 7,12-dimethylbenz[a]anthracene (DMBA)-induced skin cancer in NTT 3T3 mice fibroblast cells [123]. A study reported that kappa-carrageenan extracted from *Hypnea musciformis* was able to inhibit the proliferation of the human neuroblastoma (SH-SY5Y) and human breast cancer (MCF-7) cell lines [124]. High molecular weight (600–800 kDa) and low molecular weight (100–300 kDa) chitosan were tested as anti-carcinogenic in MCF-7, HeLa, and Saos-2 cell lines. The results revealed that both types of chitosan had a higher cytotoxic effect on the cancerous cell lines than on fibroblast-derived foreskin cells (blank sample) [125]. Palhares et al. conducted a study where the anti-tumour properties of chondroitin sulphate (CS), extracted from *Litopenaeus vannamei* shrimp, were examined. It was observed that CS had an anti-proliferative and anti-angiogenic effect in murine melanoma cells (B16F10), reducing their migration and their content in melanin and TNF-α [126]. At last, relative to exopolysaccharides, Abdelnasser et al. reported that EPS retrieved from four *Bacillus* sp. strains inhibited the proliferation of the hepatocellular carcinoma (HepG2) cell line. In particular, EPS-6 MW 43 kDa produced by *Bacillus megaterium* retrieved from the Mediterranean Sea presented the highest cytotoxicity among all the strains tested for IC50 (218 μg/mL) against HepG2 cells. This way, the studied EPSs can be regarded as promising anti-cancer drugs [127].

**Table 3:** Summary of studies concerning the anti-cancer activity of marine polysaccharides.

Bioactive Polysaccharides	Polysaccharide Source	MW	Models	Dose, Period	Experimental Method	Results	Ref.
Alginate	_	_	Breast cancer MCF-7 cell lines	6.25–50 μg/mL	3-[4,5-dimethylthiazol-2-yl]-2,5 diphenyl tetrazolium bromide (MTT) assay	Ca-alginate-based AGN-loaded beads decreased MCF-7 cell viability	[119]
Fucoidan	*Sargassum* *cinereum*	_	Colon cancer cell line Caco-2	10–1000 μg/mL	MTT assay	Inhibited growth of Caco-2 cells	[122]
Laminarin	*Turbinaria* *ornata*	689–2634 Da	Retinoblastoma Y79 cancer cells	An array of concentrations	MTT assay	Inhibited growth of Y79 cells	[120]
Laminarin	_	_	HepG2 cells	10–40 μM	MTT assay	Inhibited autophagy; Induced apoptosis	[121]
Agar	*Laminaria* *digitata*	_	DMBA-induced skin cancer mice	60–120 mg/mL, 63 days (in vivo study)	In Vivo Anticancer Assay	Normalised food intake, water intake, body weight	[123]
			NTT 3T3 mice fibroblast cells	An array of concentrations (in vitro study)	MTT Assay	Inhibited tumour development; Inhibited growth of NTT 3T3 cells	
Agaro-oligosaccharides	_	_	HCT-116 cells	10–100 µg/mL (MTT assay)	MTT Assay	Inhibited the growth of HCT-116 cells	[128]
				0–100 µg/mL (DAPI assay)	40,6-Diamidino-2-Phenylindole (DAPI) staining assay	Induced apoptosis	
ʎ Carrageenan Oligosaccharides	*Kappaphycopsis cottonii*	2 kDa	BGC-823 cells BALB/c mice	1–20 μg/mL	Cell counting kit-8 (CCK-8) assay Annexin V/PI test; Neutral red uptake assay; In vivo phagocytosis assay	Increased TNF-α and IFN-γ levels; Induced apoptosis; Improved spleen and thymus indexes; Inhibited the growth of BGC-823-derived tumours	[129]
Chitosan	_	600–800 kDa and 100–300 kDa	MCF-7, HeLa and Saos-2 cells	0.25–4 mg/mL	MTT assay	Inhibited cell proliferation in all cell lines	[125]
				1–2 mg/mL	Annexin V/PI test	Induced apoptosis and necrosis	
Chondroitin sulphate	*Litopenaeus vannamei*	_	Murine melanoma cells (B16F10)	25–100 μg/mL	Cell death, colony formation, wound healing, *Transwell* migration, and Matrigel endothelial cell tube formation assays; TNF-α quantification	Reduced tumour colony formation, cell migration, and tubular structure formation; Decreased melanin and TNF-α levels	[126]
GAG	*Oreochromis niloticus*	_	HTC and SH-SY5Y cells	1–100 μg/mL	MTT assay	Inhibited growth of HTC and SH-SY5Y cell lines	[130]
EPS	*Bacillus* sp.	37.6–51.9 kDa	HepG2 cells	125–1000 μg/mL	Neutral red uptake assay	Cytotoxic activity towards HepG2 cells	[127]

### 3.4. Regulation of Gut Microbiota

The gastrointestinal tract microbiota consists of a significant variety of microorganisms, mostly belonging to two different bacteria phyla, *Firmicutes* (contains classes such as *Bacilli*, *Clostridia*, *Erysipelotrichaceae*, and *Thermolithobacteria*) and *Bacteroidetes* (includes the genera *Bacteroides*, *Prevotella*, and *Porphyromonas*) [110,131,132]. Whilst Firmicutes typically have multiple carbohydrate metabolism enzymes that promote the metabolism of macronutrients, Bacteroidetes mostly break down complex glycans. Typically, an increased ratio of Firmicutes/Bacteroidetes is often associated with obesity and other metabolic dysfunctions, whilst a lower ratio is normally linked to a healthier organism [110]. The gut microbiota is extremely susceptible to factors such as diet, antibiotics, and physical activity [133]. Diet plays a crucial role in promoting symbiosis between bacterial communities and the human organism. Consumption of fibre-rich foods is highly beneficial, as it stimulates the bacterial production of short-chain fatty acids (SCFAs), which have been reported to have anti-cancer and anti-inflammatory effects [121,134]. The standard Western diet is typically high in fat and low in natural fibre, which inevitably alters the composition of the microbiota and its interactions with the host. These disturbances in gastrointestinal microbiota may lead to the development of disorders such as intestinal bowel disease, cancer, obesity, cardiovascular disease, and other metabolic dysfunctions [133,134]. For prevention, the reliance on natural products with bioactive ingredients has significantly increased, namely functional foods of marine origin. In recent years, researchers have studied the correlation between the ingestion of marine-derived bioactive compounds, such as polysaccharides, and the improvement of conditions associated with metabolic syndrome [110]. Characteristics such as the branching of the polysaccharide structure and their glycosidic linkages have been shown to promote SCFA production and benefit overall gut health [110,132]. Some examples of polysaccharides with this effect are shown in Table 4.

One study evaluated the impact of alginate oligosaccharides on C57BL/6J mice fed with a high-fat diet. They found that supplementation with these oligosaccharides improved lipid metabolism, reduced triglyceride (TG) and low-density lipoprotein (LDL-C) levels, and inhibited lipogenesis. It also reduced glucose levels during fasting while increasing insulin levels and reducing the expression of IL1β and CD11c inflammatory markers. Finally, these oligosaccharides impacted the microbiota by promoting the growth of species such as *Akkermansia muciniphila*, *Lactobacillus reuteri*, and *Lactobacillus gasseri*. The authors concluded that alginate oligosaccharides can be used as nutraceuticals to prevent metabolic disorders [134]. Alginates have also been reported to have a water-retaining capacity that leads to an increase in faecal bulk, thus contributing to bowel regularity and overall digestive health [14]. Tang et al. investigated the effects of fucoidan, derived from *Laminaria japonica*, on cyclophosphamide (CTX)-induced disruption of the intestinal microbiota in mice. Fucoidan was shown to significantly reverse the damage caused to the gut microbiota, inhibiting *Erysipelotrichia*, *Peptostreptococcaceae*, and *Faecalibaculum* bacteria. On the other hand, it increased the levels of important bacteria such as *Lactobacillaceae*, *Rikenellaceae*, *Gastranaerophilales*, and *Cyanobacteria* [135]. Fucoidan has also demonstrated its capacity to increase secondary bile acids production, such as Nor-DCA, TLCA, and β-UDCA, and to regulate the expression of the bile acid receptors FXR and TGR5. In this way, this polysaccharide can regulate lipid metabolism and contribute to the regulation of the intestinal microbiota [136]. A laminarin-type *β*-(1→3)-glucan named SHNP has also shown the ability to regulate the gut microbiota by influencing the bacterial content. An in vitro fermentation test with human faecal microbiota registered an increase in SCFA levels, particularly acetic acid and propionic acid [137]. Zhang et al. evaluated the effect of the oligosaccharide derived from agar neoagarotetraose (NA4) on obese mice and the gut microbiota’s role. The results indicated that this oligosaccharide could rejuvenate the intestinal microbiota and increase the secretion of SCFAs. Consequently, this oligosaccharide could be an interesting prebiotic supplement that could be used to prevent and alleviate obesity caused by a high-fat diet through the regulation of gastrointestinal microbiota [138]. A similar finding, but with respect to k-carrageenan, was reported by Wang et al. when they analysed the effects of this polysaccharide in high-fat diet-induced obese mice. The composition of the gut microbiota was assessed by the 16S rRNA sequencing of the colonic contents of the mice, and it was shown that k-carrageenan did indeed alter it. The Firmicutes/Bacteroidetes ratio in the mice supplemented with the polysaccharide was lower when compared to the mice only fed with a high-fat diet, thus indicating the effectiveness of this polysaccharide in reducing obesity by altering the gastrointestinal microbiota [139]. Chitin derivatives, such as chitosan and chitooligosaccharides, have also been tested for their potential as intestinal microbiota regulators in dextran sodium sulphate (DSS)-induced ulcerative colitis (UC) mice. These compounds were shown to restore intestinal microbiota homeostasis, promote the synthesis of SCFAs, and recover the intestinal barrier functionality of the mice. As a result, these compounds may also be used as supplements in the food industry to contribute to the treatment of ulcerative colitis [140]. A sulphated glycosaminoglycan (SBSG) derived from the swim bladder of *Aristichthys nobili* was analysed regarding its prebiotic capabilities and effect on arsenic-induced intestinal damage. The researchers found that SBSG behaved as a prebiotic by resisting degradation in the upper digestive system and promoting the growth of beneficial bacteria. In addition, this compound significantly improved the pathological damage of the jejunum and colon caused by arsenic [141]. One other study evaluated the possibility of using EPS produced by LAB as a prebiotic by fermentation of the EPS by faecal microbiota in a continuous culture system. It was concluded that the EPS produced by *Weissella cibaria* was selectively utilised by *Bifidobacteria* and *Lactobacilli*, promoting their growth and activity in the colon. EPS also provided sustained stimulation for the growth of *Bifidobacteria*, unlike most common commercialised prebiotics, whose effects are shorter-lived, hence demonstrating its capability as a prebiotic [87].

**Table 4:** Summary of studies concerning regulation of gut microbiota of marine polysaccharides.

Bioactive Polysaccharides	Polysaccharide Source	MW	Models	Dose, Period	Experimental Method	Results	Ref.
Alginate oligosaccharide	**_**	_	HFD-fed C57BL/6J mice	5 g/100 g mice feed, 10 days	Histopathological examination; Gut microbiota analysis; SCFAs, serum biochemical analysis; Insulin and endotoxin quantifications	Reduced fat accumulation and inflammation in the liver; Regulated intestinal flora composition; Reduced TG and LDL-C levels; Increased SCFAs levels	[134]
Fucoidan	*Laminaria* *japonica*	250 kDa	CTX treated mice	20 or 40 mg/kg BW, 19 days	Histopathological examination; Gut microbiota analysis	Reversed the damage in the spleen and thymus; Regulated intestinal flora composition	[135]
Fucoidan	*Laminaria* *japonica*	14.6 kDa	Diabetes mellitus (DM) model mice	100 mg/kg, 13 weeks	Serum biochemical analysis; Histopathological examination; Gut microbiota analysis; Non-targeted metabolomics and bile acid analysis	Reduced TG, total cholesterol (TC), and (LDL-C); Reduced liver damage; Regulated intestinal flora composition; Modulated bile acid content	[136]
SHNP (laminarin-type *β*-(1→3)-glucan)	*Sargassum henslowianum*	8.4 kDa	Faecal inocula from healthy individuals	20 mg/mL (in vitro study)	In vitro fermentation of human faecal microbiota; SCFA analysis	Regulated intestinal flora composition; Increased SCFA levels	[137]
Neoagarotetraose, NA4 (from agar)	_	630 Da	HFD-fed C57BL/6 mice	200–800 mg/kg/day, 12 weeks	Biochemical assays; Histochemical staining SCFA analysis; Gut microbiota analysis; Real-time quantitative PCR	Reduced insulin resistance and improved glucose tolerance; Reduced fat deposition and ballooning in hepatocytes; Increased SCFA levels; Regulated intestinal flora composition; Reversed damage to the intestinal barrier	[138]
κ Carrageenan	_	_	HFD-fed C57BL/6J mice	0.2–1% (*w*/*w*), 8 weeks	Oral glucose tolerance test; Serum biochemical analysis; Histopathological examination; RT-qPCR; SCFA analysis	Reduced weight gain; Reduced TG, TC, and LDL-C; Reversed damage to the intestinal barrier; Regulated intestinal flora composition; Increased ratio of p-AMPK/AMPK; Increased SCFA levels	[139]
Chitin derivatives	_	20–50 kDa	DSS-induced UC C57BL/6 mice	An array of concentrations 14 days	Disease activity index (DAI) analysis; Enzyme-linked immunosorbent assay (ELISA); RT-qPCR; SCFA analysis	Reduced weight loss; The colonic stratified structure remained mostly intact; Reversed damage to the intestinal barrier; Regulated intestinal flora composition; Increased SCFA levels	[140]
Chitosan	_	_	Grass carp infected with *F. columnare* Grass carp liver cells (L8824)	0.03 g/1000 g feed (in vivo study), 56 days	Serum biochemical analysis; Histopathological examination; 16s rRNA gene; sequencing	Reduction in mortality and increased immunity; Improved intestinal flora diversity	[142]
SBSG	*Aristichthys* *nobilis*	54 kDa	In vitro	0–2 mg/mL (in vitro study)	Simulated in vitro digestion and in vitro evaluation of prebiotic activity	SBSG does not degrade in the upper gastrointestinal tract; Regulated intestinal flora composition	[141]
			C57BL/6J mice	50–200 mg/kg (in vivo study) 38 days	Histopathological examination	Reversed the damage in the jejunum	
EPS	Lactic acid bacteria	_	HT-29 epithelial cells	0.001–1% (*w*/*v*)	Digestibility assay; Growth and EPS use by probiotic bacteria; Fermentation through a continuous culture system; SCFA analysis	Growth of probiotic bacteria and inhibition of food-borne pathogens; Production of vitamins B1, B2, and folic acid; Increased SCFA levels	[87]

### 3.5. Anti-Obesity and Anti-Diabetic Activity

In recent years, the prevailing sedentary lifestyle and poor dietary choices have led to a significant increase in conditions such as obesity and diabetes within our society. These diseases are intricately connected, as the risk of developing type 2 diabetes is directly correlated to an increase in body fat [14,143]. Type 2 diabetes consists of generalised insulin resistance and disruption of β cells functioning as insulin secretors [144]. The standard approach to the treatment of this dysfunction is the prescription of hypoglycaemic pharmaceuticals or the injection of insulin. Nonetheless, these treatments are not always efficient and may even prove harmful. As a result, nutraceutical and functional foods have been emerging as a possible supplementary treatment to diabetes, as is the case of marine polysaccharides [28]. Marine polysaccharides have generally revealed anti-diabetic effects through their capacity to behave as dietary fibre, explained by their gel-forming ability and glycosidic bonds (i.e., β-(1→3), β-(1→4), and β-(1→6) linkages that cannot be digested by human enzymes) and inhibition of α-glucosidase [14,143]. The inhibition of the activity of α-glucosidase is of particular interest as this enzyme, alongside α-amylase, is responsible for the hydrolysis of carbohydrates, which leads to a decrease in post-prandial glucose levels [112,145]. There are countless reports on the anti-obesity and anti-diabetic effects that different marine polysaccharides have displayed (Table 5).

One study explored the potential anti-obesity effects of low-molecular alginate derived from *Laminaria japonica*. The authors noticed a reduction in weight gain and accumulation of fat in the liver and epididymal adipose tissue of mice fed a high-fat diet. Additionally, low-molecular-weight alginate also promoted the alteration of gut microbiota, which inevitably lead to the improvement of the metabolic syndrome [143]. Wu et al. studied the anti-obesity effects of fucoidan extracted from the algae *Sargassum fusiforme* on a high-fat diet and streptozotocin-induced type 2 diabetes mice (T2DM). Fucoidan was revealed to have greatly reduced glucose and lipid levels in the bloodstream alongside the mice’s appetite. Meanwhile, the damaged adipose, liver, and heart tissues showed significant improvement. For this reason, this study concluded that fucoidan may be used as a functional food or nutraceutical to reduce the problems associated with type 2 diabetes symptoms and other related issues [146]. Jayapala et al. studied the anti-diabetic potential of laminarioligosaccharides obtained from laminarin using α-amylase and α-glucosidase inhibition assays. The results confirmed the anti-diabetic activity, as the oligosaccharides displayed a maximum α-amylase and α-glucosidase inhibition of 32.2% and 58.8%, respectively, at a laminarin concentration of 30 μg [147]. A preclinical study using in vivo models also focused on investigating the anti-obesity properties of laminarin. This study confirmed that laminarin could slow diet-induced obesity and increase catabolic carbohydrate enzymes, even if only during the period of laminarin administration. Thus, the authors suggested that this polysaccharide could be useful as a supplement that would have to necessarily be consumed regularly to ensure the prevention and management of obesity [148]. Agaropectin-derived oligosaccharides have also been proven to have anti-diabetic activities, as they can improve insulin sensitivity and glucose metabolism [149].

Obesity is associated with cardiovascular disease and risk factors such as high levels of cholesterol and blood pressure, so compounds that target these factors may also help address obesity [150]. Carrageenans have demonstrated their capability to reduce plasma cholesterol, as it can increase intestinal viscosity, thus interfering with its absorption. Furthermore, the fermentation of carrageenan also hinders the biosynthesis of cholesterol. All this suggests that carrageenan can be used as a supplement to reduce cholesterol levels [52]. Low-molecular-weight carrageenan has also been proven to have anti-hyperglycaemic and anti-hyperlipidaemic activities in mice fed a high-fat diet. The authors observed a decrease in postprandial blood glucose, total cholesterol, triglyceride, and LDL-C levels, as opposed to high-density lipoprotein (HDL-C), whose levels increased [151]. Similarly, Preez et al. also showed the potential use of carrageenan to help in the treatment of metabolic syndrome [53]. One other way for polysaccharides to contribute to the treatment of obesity and diabetes is by being used as delivery systems, as is often the case with chitosan. Recent studies have focused on drug delivery using modified chitosan, as it is more bioavailable, thus enhancing the uptake into target cells. Chitosan derivatives may be used in the form of nanoparticles, capsules, and hydrogels depending on the desired functionality [134]. This type of drug delivery system was tested by Chen et al. In this study, chitosan-thioglycolic acid (CT) was first developed, and it showed mucoadhesive properties and inhibited nutrient uptake in the gastrointestinal tract. Subsequently, hesperidin, a Chinese herb that promotes weight control, was encapsulated in CT, and the results of the studies in mice indicated that it controlled weight gain. Moreover, it also limited adipose tissue accumulation due to the interference with nutrient absorption; therefore, it can be a promising strategy to tackle obesity [152]. Glycosaminoglycans may also display anti-obesity activities by modulation of enzymes and lipid metabolism overall. In particular, Li et al. investigated this phenomenon in chondroitin sulphate oligosaccharides with different molecular weights collected from the skate *Raja pulchra*. The in vitro study showed that CSs inhibited lipase activity, lipid accumulation in 3T3-L1 preadipocyte cells, and intestinal absorption of triglycerides. Moreover, the study conducted in male HFD C57B/6 J mice determined that CSs did indeed regulate general body weight gain as well as liver and adipose tissue weight gain. The authors concluded that CS molecular weight affected its activities, as higher MW CSs were stronger lipase inhibitors, whilst low MW CSs were more successful in suppressing adipocyte lipid accumulation [153]. Lastly, exopolysaccharides have also been found to exert an anti-obesity effect. In a study, EPS had a lipase-inhibitory activity of 95.3% at a concentration of 1000 μg/mL, and it inhibited α-amylase (IC_50_ 31.49 μg/mL) and α-glucosidase (IC_50_ 6.48 μg/mL). Thus, the studied EPS has proven to have promising in vitro anti-diabetic properties, surpassing those of Acarbose, the pharmaceutical used as a control [154].

**Table 5:** Summary of studies concerning anti-obesity and anti-diabetic activities of marine polysaccharides.

Bioactive Polysaccharides	Polysaccharide Source	MW	Models	Doses	Experimental Method	Results	Ref.
Alginate	*Laminaria* *japonica*	110 kDa	HFD-fed BALB/c mice	0.3%, 11 weeks	Serum biochemical analysis; Histopathological examination; Faecal transplantation; SCFA analysis	Reduced TG, TC, andLDL-C levels; Reduced weight gain, fat accumulation in the liver, and epididymal adipose tissue; Regulated intestinalflora composition; Increased SCFA levels	[143]
Fucoidan	*Sargassum* *fusiforme*	205.8 kDa	HFD- and streptozotocin (STZ)-induced T2DM mice	100 mg/kg; BW/day, 1 month	Intraperitoneal glucose tolerance test (IPGTT); Serum biochemical analysis; Histopathological examination; 16s rRNA gene sequencing	Decreased polydipsia and polyphagia; Reduced TG, TC, and LDL-C levels; Reduced weight gain, fat accumulation in the liver, heart, and adipose tissue; Regulated intestinal flora composition	[146]
Laminarioligosaccharides	*Laminaria* *digitata*	_	In vitro	10–30 μg	α-Amylase and α-glucosidase inhibition assay	Inhibited α-amylase and α-glucosidase	[147]
Laminarin	_	_	HFD-fed BALB/c mice	1%, 6 weeks	16s rRNA gene sequencing; Microbial community analysis; Metagenome sequences analysis	Decreased weight gain Regulated intestinal flora composition	[148]
Agaropectin derived oligosaccharides	*Gloiopeltis* *furcata*	1500 Da	HepG2 cells	An array of concentrations	Glucose consumption and insulin sensitivity assays; TG, TC, and superoxide dismutase (SOD) assays	Enhanced insulin sensitivity and glucose metabolism; Decreased accumulation of lipids and improved lipid metabolism	[149]
Carrageenan	*Eucheuma* *spinosum*	1398 Da	HFD-fed Wistar rats	1–3%, 30 days	Serum biochemical analysis	Reduced TG, TC, and LDL-C levels; Decreased weight gain and food availability; Increase in faecal moisture	[151]
ι- Carrageenan	*Sarconema* *filiforme*	_	HFD-fed Wistar rats	5% (~1.05 g/day), 4 months	Serum biochemical analysis; Glucose consumption and insulin tolerance assays; Microbial community analysis	Decreased weight gain, food intake, systolic blood pressure, and TC; Regulated intestinal flora composition	[53]
Thiolated chitosan	_	_	IEC-6 cells	6 mg/mL (in vitro study)	In vitro mucus adhesion test	Exhibited mucoadhesive properties	[152]
			HFD-fed male C57BL/6 mice	250 mg/kg/ day (in vivo study), 8 weeks	Oral glucose tolerance test (OGTT) and intraperitoneal glucose tolerance test (IPGTT); In vivo adhesion test; Serum biochemical analysis	Reduced lipid accumulation; Reduced TG, TC, and LDL-C levels; Controlled weight gain	
Chondroitin sulphate oligosaccharides	*Raja pulchra*	0.46–250 kDa	3T3-L1 preadipocyte cells	An array of concentrations (in vitro study)	In vitro pancreatic lipase assay; Triglyceride E-test kit	Inhibition of lipase activity, absorption of TG, and lipid accumulation	[153]
			HFD-fed male C57BL/6 mice	50 mg/ 5 mL/kg/day (in vivo study), 8 weeks	Serum biochemical analysis	Decreased full body, liver, and adipose tissue weight	
EPS	*Streptomyces* *vinaceusdrappus*	51 kDa	In vitro	1.95 to 1000 μg/mL	Inhibition of lipase enzyme assay; α-Amylase and α-glucosidase inhibition assay	Inhibited lipase enzyme, α-amylase, and α-glucosidase	[154]
PJ1-1	*Penicillium* *janthinellum* *N29*	10.24 kDa	In vitro	0.08–5.00 mg/mL (in vitro study)	α-glucosidase inhibitory assay	Inhibited α-glucosidase	
			Type 2 diabetes C57BL/6J male mice	100–400 mg/kg/day, 35 days (in vivo study)	Serum biochemical analysis of mice; Glucose consumption and insulin sensitivity assays	Decreased lipid accumulation; Reduced TG, TC, and LDL-C levels, and increased HDL; Increased insulin sensitivity and glucose tolerance and reduced blood glucose level	[155]

## 4. Functional Properties of Marine-Derived Polysaccharides

Beyond the biological properties of marine-derived polysaccharides, the functional ones are also of great importance, as they are essential to the polysaccharides’ applicability and versatility in the food and nutraceutical industries.

Depending on their structure, ionic type, pH, and temperature, polysaccharides can form gels when in contact with water. The polymer forms a three-dimensional structure through the establishment of intermolecular interactions, such as hydrogen and ionic bonds, as well as hydrophobic interactions [155]. As discussed in the section on alginate, this compound is an example of a marine polysaccharide that displays a strong gelling capacity, similar to carrageenan and agar. In fact, agar was the first phycocolloid ever to be utilised as a food additive and is also considered the most efficient one. This is due to the extraordinary gelling power that makes it so that a 1% concentration is enough to be effective. In addition, its heat resistance and reversible gelling abilities are also exceedingly useful in the confectionery sector, for example [15,50,52,156,157].

Its other functional properties are as a thickener and stabiliser. Thickeners are compounds that entangle their chains, creating a resistance in the fluid to a change in shape. A stabiliser behaves similarly, being able to confine oil droplets, hence providing lasting stability to emulsion systems. Chitosan is an example of a polysaccharide that acts as an emulsifier due to its structure being partially deacetylated; thus, it might provide a more thorough stabilisation [61,62,157]. Carrageenan has the particularity of being able to react with proteins, which is why it is often used in drinks, such as milk, to stabilise its proteins [17,157].

These polysaccharides can also act as fibres, displaying a water-binding ability that depends on their chemical structure, porosity, and ionic form, among other factors. This property plays a crucial role in ensuring the polysaccharide’s effectiveness as an anti-obesity agent and for its ability to regulate food texture.

One other characteristic that is especially useful in the nutraceuticals and the food industry is the potential to replace fat in sausages, for example. Some polysaccharides can bind to the additional water meant to replace the fat, upholding the original texture [156].

Marine polysaccharides are, therefore, a frequently used tool in the food industry to manipulate the texture, viscosity, and emulsion stability of food products. Several of them have already been granted the status of GRAS, as previously discussed. Examples include their roles as gluten substitutes in baked goods and the stabilisation of icings, sauces, ketchup, yoghurt, and cheese [3,9,15,156].

## 5. Applications of Marine Polysaccharides in Nutraceuticals

As mentioned above, marine polysaccharides can be used in the nutraceutical field in a variety of ways due to both their biological and functional properties. They may act as active ingredients contributing to a certain health benefit, or they can have a structural role and improve the formulation of a product or even facilitate the transportation of an active compound, improving its bioavailability.

Over the past few years, research in the nutraceutical sector has grown significantly, driven by the increasing demand for a wide variety of supplements. Many of these studies have tested supplements of marine polysaccharides in clinical trials with very promising results [5,12,13].

One of these studies focused on investigating the efficiency of alginate supplements with different mannuronic/guluronic (M/G) ratios in promoting satiety and thus contributing to weight loss. It was observed that the supplements with a lower M/G ratio had the most success in satiating the eight participants [157]. Moraru et al. also conducted a clinical trial where obese and overweight participants were given chitosan supplementation for 52 weeks. The researchers achieved promising results, as there was a reduction in body weight, a decrease in blood pressure, and an improvement in the serum lipid profile of the individuals [158]. One other study intended to evaluate the effects of fucoidan supplementation on *Helicobacter pylori*-infected patients. The clinical trial showed that this supplementation had a positive effect on the gut dysbiosis caused by the infection and its treatment [159]. These studies, along with the ones presented in Table 1, Table 2, Table 3, Table 4 and Table 5, suggest that regular supplementation with marine-derived polysaccharides will bring positive health effects, contributing to the improvement of numerous medical conditions. Nonetheless, reaching the final commercialisation step is still fairly challenging for some of these compounds. This is likely due to the bureaucracy involved as well as the production process, which has not been fully optimised, resulting in higher costs, as seen with marine exopolysaccharides [160]. There are, however, several brands that have started commercialising supplements of these compounds, particularly those that have been more thoroughly studied. Brands such as MarkNature, Life Extension, NOW, and Natural Balance are examples of companies that have invested in alginate, fucoidan, agar, and chitosan supplements.

## 6. Conclusions and Future Prospects

Marine-derived polysaccharides such as alginate, fucoidan, laminarin, agar, carrageenan, chitin/chitosan, glycosaminoglycans, and exopolysaccharides exhibit an extensive array of biological and functional properties that position them as pivotal ingredients in the nutraceutical sector. In this paper, we have focused on the biological effects that are known to be associated with these compounds, such as antioxidant, anti-inflammatory, anti-cancer, gut microbiota regulating, anti-obesity, and anti-diabetic activities. Countless studies have confirmed the positive health effects of these polysaccharides as a supplement in the treatment of certain diseases without presenting any toxicity. In some of these studies, it has been noted that derivatives of these polysaccharides had more pronounced biological effects, as was often the case with agar, possibly as a result of their greater bioavailability. Moreover, these polysaccharides also feature incredibly useful functional characteristics for both the food and nutraceutical industries, such as acting as stabilisers, emulsifiers, gelling agents, and fat replacers. All of these properties enhance their value in boosting the sensory and textural attributes of food and nutraceutical products. The synergy between bioactivity and functionality emphasises the utmost relevance of marine polysaccharides, which consist of their health benefits coupled with the improvement of the product’s formulation. This allows the products to meet consumer demands for health-promoting, natural, and sustainable ingredients.

Yet, there are still many challenges to overcome before these compounds can successfully reach the customers. Further research is needed to fully understand the mechanisms of action and potential effects of these polysaccharides on the human body. This way, well-structured clinical trials must be conducted to fully ensure their safe use by future customers. These polysaccharides are highly susceptible to structural change, for example, due to environmental factors, which leads to inconsistency that will undeniably affect the production process. A possible solution might be an investment in standardisation extraction and purification protocols to assure constant quality. The scaling-up of the production process must also be carefully considered so that the compounds retain their bioactivity whilst also taking into consideration sustainability and efficiency factors. As an example, the harvesting of marine resources must be conducted diligently to minimise disturbance to marine ecosystems. Additionally, biotechnological approaches can offer a potential means to produce exopolysaccharides through microbial fermentation. Finally, regulations must also be revised so that guidelines can be consistent, clear, and easy for the nutraceutical industry to follow.

Marine-derived polysaccharides have proven to be a sustainable, multifaceted, and scientifically relevant solution to many of the health problems troubling today’s society. Interdisciplinary collaboration between researchers, companies, and legislators is vital in order to make meaningful progress in the practical application of these compounds within the global nutraceutical market.

## Data Availability

No new data were created or analyzed in this study.
